# MUSIC AND QUALITY OF LIFE IN OLDER ADULTS

**DOI:** 10.1093/geroni/igad104.0791

**Published:** 2023-12-21

**Authors:** Lisa Lehmberg, Victor Fung

**Affiliations:** University of Massachusetts Amherst, Amherst, Massachusetts, United States; University of South Florida, Tampa, Florida, United States

## Abstract

Research studies have indicated that music is an important contributor to older adults’ quality of life. In this segment, the presenter will share overarching observations from their original research and the literature regarding music making, listening, creating, and activities involving movement to music in relation to facets of quality of life, such as identity, social connections, enjoyment, and self-understanding and expression. These studies were done in a range of contexts (e.g., retirement communities and senior centers), formats (small and large groups, participatory and presentational), and groups that engage in different musical styles (e.g., classical and vernacular). Music has been shown to be critical to the quality of life of those who participate. However, additional research is needed with diverse populations, including those who are homebound, homeless, in supervised care (e.g., assisted living, nursing homes, or hospice care), as well as older adults of varying ages and national origin.

